# The one-key-to-two-doors role of lipidomics: plasma lipidome, cardiovascular risk and statin usage

**DOI:** 10.1016/j.jlr.2025.100802

**Published:** 2025-04-14

**Authors:** Anatol Kontush

**Affiliations:** National Institute for Health and Medical Research (INSERM), UMRS 1166 ICAN, Faculty of Medicine Pitié-Salpêtrière, Sorbonne University, Paris, France

Statin therapy stands out as a remarkably effective and cost-efficient approach for both the prevention and treatment of cardiovascular disease. Statins significantly reduce cardiovascular disease events by inhibiting HMG-CoA reductase, the key enzyme in cholesterol production. This action lowers circulating levels of LDL-cholesterol, a major risk factor for cardiovascular disease.

Recent research indicates that lipid metabolism, beyond just LDL-cholesterol, plays a central role in the development and progression of cardiovascular disease (1, 2, 3). Circulating concentrations of various lipid species can improve prediction of the onset of cardiovascular disease on top of traditional cardiovascular risk, including LDL-cholesterol (4, 5). However, statins induce major alterations of lipid metabolism that can markedly modify circulating lipidome beyond their impact on LDL-cholesterol (6). These effects primarily include reduction in plasma phospholipids and sphingolipids. For example, rosuvastatin significantly decreases the concentrations of various phospholipids, including phosphatidylcholine, lysophosphatidylcholine, and phosphatidylinositol, as well as of total and individual plasma sphingolipids in a dose-dependent manner when taken by subjects presenting with metabolic syndrome (7). These lipidomic alterations occur in parallel to the LDL-cholesterol-lowering effect of statins and may contribute to their pleiotropic effects, including anti-inflammatory and antiatherosclerotic properties. Furthermore, statins decrease plasma cholesteryl ester transfer protein (CETP) activity in both normolipidemic individuals and patients with hyperlipoproteinemia, raising HDL-cholesterol and reducing triglycerides (8).

Statins thereby cause changes in lipoprotein metabolism in the circulation, which are central to their beneficial effects. As a result, these medications markedly confound the relationship between lipid species and cardiovascular disease risk. When investigating the relationship between the lipidome and cardiovascular disease, it is therefore essential to account for statin usage to prevent misleading conclusions. Although knowledge of statin usage is important to evaluate the risk, the effect of adjusting for statin usage in these analyses has not been thoroughly investigated. Furthermore, many studies face challenges due to inadequate or low-quality data on medication usage (9). Practical issues often arise in real-world settings due to the frequent lack of information regarding statin use. The question of how to take into account statin usage hampers the applicability of the lipidomic risk scores to clinical populations.

To address this important issue, Yi *et al.* investigated the impact of statin treatment on the circulating lipidome through the Long-term Intervention with Pravastatin in Ischemic Disease (LIPID) trial, a large, randomized, placebo-controlled study, with measurements taken in 4,991 participants before and 12 months after starting the drug (10). The study confirmed previous findings that statin treatment generally leads to reductions of circulating concentrations of many lipid species, including those of cholesteryl esters, phospholipids and sphingolipids, while a few showed increases. By combining data from 24,194 individuals across several studies, Yi *et al.* showed that circulating lipidomic profiles could effectively predict statin usage. Through the analysis of three extensive population datasets and a longitudinal clinical study, they established that lipidomic-based models for predicting statin use exhibit high accuracy in external validation.

The authors also analyzed a population-based cohort of 10,339 individuals to highlight biases in lipidomic studies that excluded statin usage, revealing significant increases in false positive associations with and inflated odds ratios for cardiovascular disease that could lead to erroneous conclusions about the role of individual lipid species. More specifically, they observed that adjusting for statins enhanced the strength of associations for many lipids and identified 303 potential false positives and 35 false negatives in models that did not account for statin usage. These novel results highlight the importance of including statin adjustment when studying the relationship between lipid profiles and disease.

Furthermore, the authors addressed challenges related to incomplete lipidomic data across studies. To this end, they introduced a novel re-weighted transfer method aimed at addressing a common problem faced by prediction models: the necessity for alignment of predictors between training and target datasets. To improve the transferability of lipidomic prediction models, a flexible transfer model was developed that allowed for the re-weighting of coefficients without requiring individual-level data. This approach enhanced the transferability of the models without significantly compromising accuracy. This method demonstrated comparable predictive performance to traditional ridge models in external validation. The authors provide an R package for developing and utilizing these re-weighted flexible transfer models, which can be useful from a practical point of view.

In particular, ridge regression models predicting statin usage using the lipidome from the Australian Diabetes, Obesity and Lifestyle Study (AusDiab) study involving 747 lipid species showed excellent performance in the three large-scale cohorts, achieving area-under-the-curve (AUC) values over 0.90. In contrast, when using a less detailed lipidomic dataset from the LIPID study only involving 342 lipid species, the prediction accuracy decreased significantly, with AUC values from 0.72 to 0.84. This result suggests that the models may not effectively capture statin usage impacts in the LIPID study due to its limited lipidomic data. Robustness testing indicated that reducing the number of lipid species led to lower AUC values. Comprehensive coverage of lipid species and classes is therefore crucial for accurate prediction models of statin usage.

Lipid species are influenced by a complex network of molecular pathways, leading to strong correlations among different lipid classes in lipidomic datasets. These correlations introduce redundancy, causing highly correlated lipid species to compete for inclusion in predictive models. Using a ridge penalty can help share weights among these correlated variables, allowing for the substitution of one lipid species with another without significantly affecting the model's generalizability. Additionally, when a specific lipid species is not measured, its concentration can often be predicted using a linear combination of other measured species. This flexibility is an important feature of lipidomic data that enables re-weighting of predictors while maintaining predictive accuracy. This feature is important from the analytical point of view as lipidomic and metabolomic platforms can measure up to thousands of lipid species, but each employs different analytical strategies, resulting in a limited overlap of measured variables. While the diversity of these platforms offers new research opportunities, it poses a challenge for developing broadly applicable prediction models (11).

Missing data presents another challenge for risk prediction models. In clinical settings, physicians can directly address it by ordering tests or collecting additional information. However, in research or retrospective studies, re-acquiring missing data is often not feasible. As a result, many studies have explored missing value imputation as a solution. This method can be effective when dealing with large datasets and sparse or randomly missing data. However, it is not appropriate for predicting risk for individual patients or when entire variables are missing for all subjects. Alternative approaches involve creating multiple prediction models in advance to accommodate new data. The method proposed by Yi *et al.* includes specifying the prediction model using two matrices instead of traditional beta-coefficients, allowing for the estimation of new beta-coefficients based on any subset of original variables without needing the original dataset. This approach offers considerable potential to enhance the transferability of 'omics risk prediction models.

As every milestone work, this study has several limitations, particularly regarding the inconsistent information on statin usage among the cohorts. For example, the AusDiab cohort only relied on a general questionnaire about cholesterol-lowering medications. Even more importantly, the study did not model the specific types or dosages of statins used; participants in the LIPID trial received a fixed dose of pravastatin, whereas the population cohorts likely included various types and dosages. Modern statins are more effective at lower doses, and clinical practice typically adjusts dosages to achieve target LDL-cholesterol levels. In addition, the impact on the plasma lipidome can vary between hydrophilic and hydrophobic statins, with hydrophilic statins showing more pronounced effects in some studies (12). Future research should explore whether the type and dosage of statins significantly influence lipidomic outcomes. Finally, while the use of statin combinations with other lipid-modifying drugs is becoming more common in clinical practice, the cohorts studied by Yi *et al.* predominantly received individual statin treatments—a strategy that was prevalent in the 1990s when these studies began. Therefore, it would be valuable to explore how the plasma lipidome is affected by the combination of statins with other medications.

One can safely conclude that adjusting for statin usage is essential when evaluating the relationship between the lipidome and cardiovascular disease. Neglecting this adjustment can lead to confounding, misattribution of effects, and misleading interpretations. As statin usage markedly influences the lipidome, extending beyond mere changes in lipoprotein levels, failing to account for it results in spurious associations between lipid species and cardiovascular disease. Hence, the necessity of incorporating statin usage into analyses. However, it is common for clinical studies, particularly those involving large population cohorts, to encounter missing or unreliable data regarding statin use.

From a practical point of view, it has been increasingly difficult to reliably assess statin usage in large population cohorts. Although individual metabolites of major statins can be measured in the circulation by LC/MS-MS as fingerprints of their parental molecules (13), this methodology is costly, time-consuming and hardly applicable in the clinical routine. Reliable evaluation of statin usage using plasma lipidomic data, which are primarily employed to calculate cardiovascular risk through the lipidomic risk scores, allows opening two doors with a single key (Fig. 1). Such adjustment for statin usage can be further developed to identify lipidomic fingerprints of other lipid-lowering medications, generalizing this strategy.

**Fig. 1:**
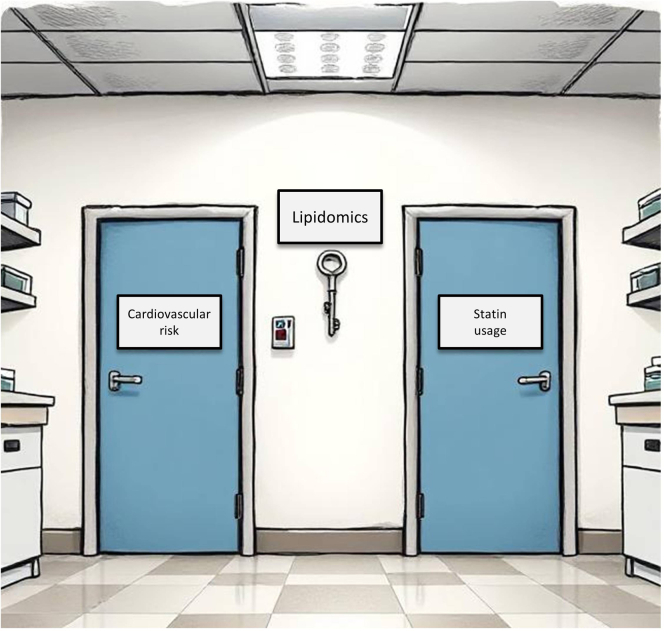
One-key-to-two-doors role of lipidomics. Assessing statin usage in large populations is challenging. Although individual statin metabolites can be quantified using LC/MS-MS, this method is costly and impractical for routine clinical application. In contrast, evaluating statin usage through plasma lipidomic data—an approach that is gaining traction for calculating cardiovascular risk—provides a more feasible solution. This method not only enhances the assessment of cardiovascular risk but can also be effectively adapted to identify statin users within large populations.

In this context, the findings of Yi *et al.* that lipidomic data can effectively predict statin usage are of immediate clinical relevance. Their innovative approach can be applied in order to improve risk prediction across large cohorts. The flexible transfer model developed by the authors demonstrates performance comparable to ad-hoc models, thereby considerably enhancing the transferability of prediction models without compromising accuracy. This approach represents an important advancement that can enhance the clinical application of lipidomics.

## Conflicts of interests

The author declares that there are no conflicts of interest with the contents of this article.
